# Case Report: Transfusion-associated graft-versus-host disease in severe combined immunodeficiency

**DOI:** 10.3389/fimmu.2025.1708366

**Published:** 2026-01-12

**Authors:** Chunxue Jiang, Tingting Sun, Xuewen Xu, Wei Xu, Kai You

**Affiliations:** 1 Department of Pediatrics, Shengjing Hospital of China Medical University, Shenyang, China; 2 Department of Urology, Shengjing Hospital of China Medical University, Shenyang, China

**Keywords:** child, eosinophil, lymphocyte, severe combined immunodeficiency, transfusion-associated graft-versus-host disease

## Abstract

Transfusion-associated graft-versus-host disease (TA-GVHD) is a rare but fatal blood transfusion complication, with a mortality rate of 90-100%. Severe combined immunodeficiency (SCID) is a life-threatening primary immunodeficiency with profound cellular and humoral defects. Patients with SCID are highly susceptible to TA-GVHD. Here, we report a 4-month-old male admitted for sepsis and severe pneumonia, with pustular rash and unhealed exudative Bacillus Calmette-Guérin vaccination site. Laboratory tests showed hypogammaglobulinemia and lymphopenia. Lymphocyte subset analysis confirmed the presence of T-B+NK immunodeficiency. Mycobacterium bovis complex was detected in blood, while rifampicin-resistant Mycobacterium tuberculosis complex was identified in sputum and ascitic fluid. Whole-exome and Sanger sequencing identified a novel interleukin-2 receptor common gamma chain (IL2RG) nonsense mutation [NM_000206.3: c.865C>T, p.(Arg289Ter)]. To the best of our knowledge, this specific IL2RG mutation has not been previously reported. On the 33rd day of admission, the infant accidentally received non-irradiated leucoreduced red blood cells, then developed typical TA-GVHD manifestations including fever, hepatomegaly, rash and diarrhea, and high-resolution Human Leukocyte Antigen typing confirmed it. The parents chose to terminate treatment on the 69th day of admission, and the patient died after discharge. The dynamic evolution of clinical manifestations and laboratory tests in this patient is described, along with a review of the relevant literature. This report expands the mutational spectrum of IL2RG and reveal the reference value of peripheral blood lymphocyte and eosinophil counts for early TA-GVHD identification.

## 1 Introduction

Transfusion-associated graft-versus-host disease (TA-GVHD) is a rare complication of blood transfusion wherein donor lymphocytes in a transfused blood component mount an immunodestructive response against recipient tissues (1–6). Mortality in TA-GVHD has been estimated to be between 90-100% (1). It is manifested as fever, rash, gastrointestinal symptoms, liver injury, and pancytopenia (1, 4, 7, 8). Bone marrow aplasia and histiocytic infiltration of the intestine, spleen, and marrow are pathologic hallmarks (9). Severe combined immunodeficiency (SCID) is a primary immunodeficiency caused by genetic defects, characterized by profound defects in both cellular and humoral immunity (8, 10–12). Patients with SCID have defective delayed hypersensitivity reactions and therefore are particularly prone to being tolerant to allogeneic grafts, making them highly susceptible to TA-GVHD after transfusion (4, 10). Among SCID subtypes, X-linked SCID caused by mutations in the interleukin-2 receptor common gamma chain (IL2RG) gene is a common type, and such patients are also at high risk of TA-GVHD (10, 12).

Since clinical TA-GVHD can easily be misinterpreted as drug reactions, viral infections, or sepsis syndrome, its diagnosis may be overlooked (4, 8, 13). Here we report a case of SCID with a novel IL2RG mutation who developed TA-GVHD following transfusion of non-irradiated leucoreduced red blood cells. The dynamic evolution of clinical manifestations and laboratory tests in this patient is described, along with a review of the relevant literature.

## 2 Case description

**Figure 1:**
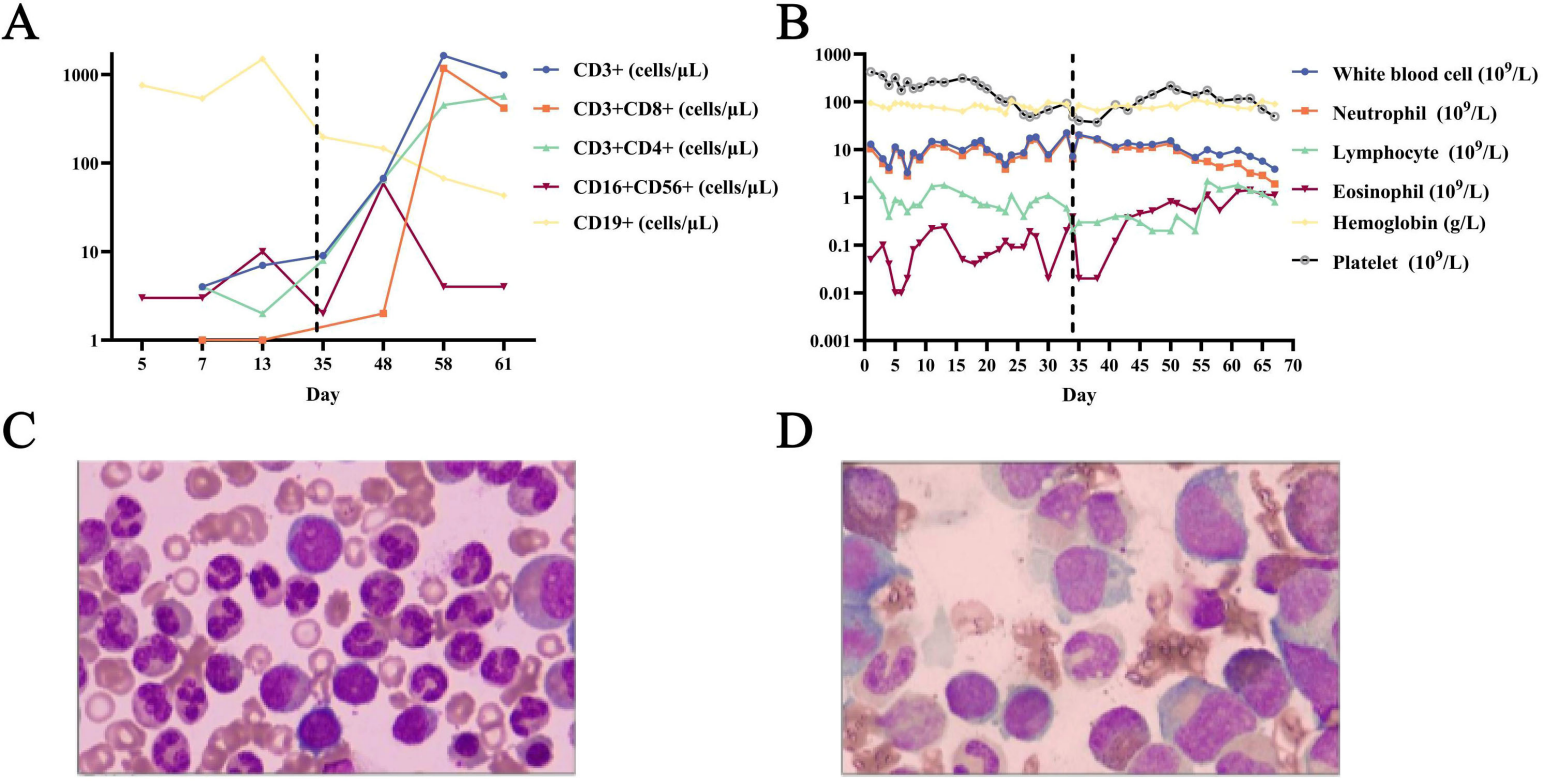
Detailed laboratory indicators and bone marrow examination. **(A)** Dynamic changes of inflammayory markers. **(B)** Dynamic change of peripheral blood lymphocytes. **(C)** Initial bone marrow examination. **(D)** Bone marrow re-examination.

**Figure 2:**
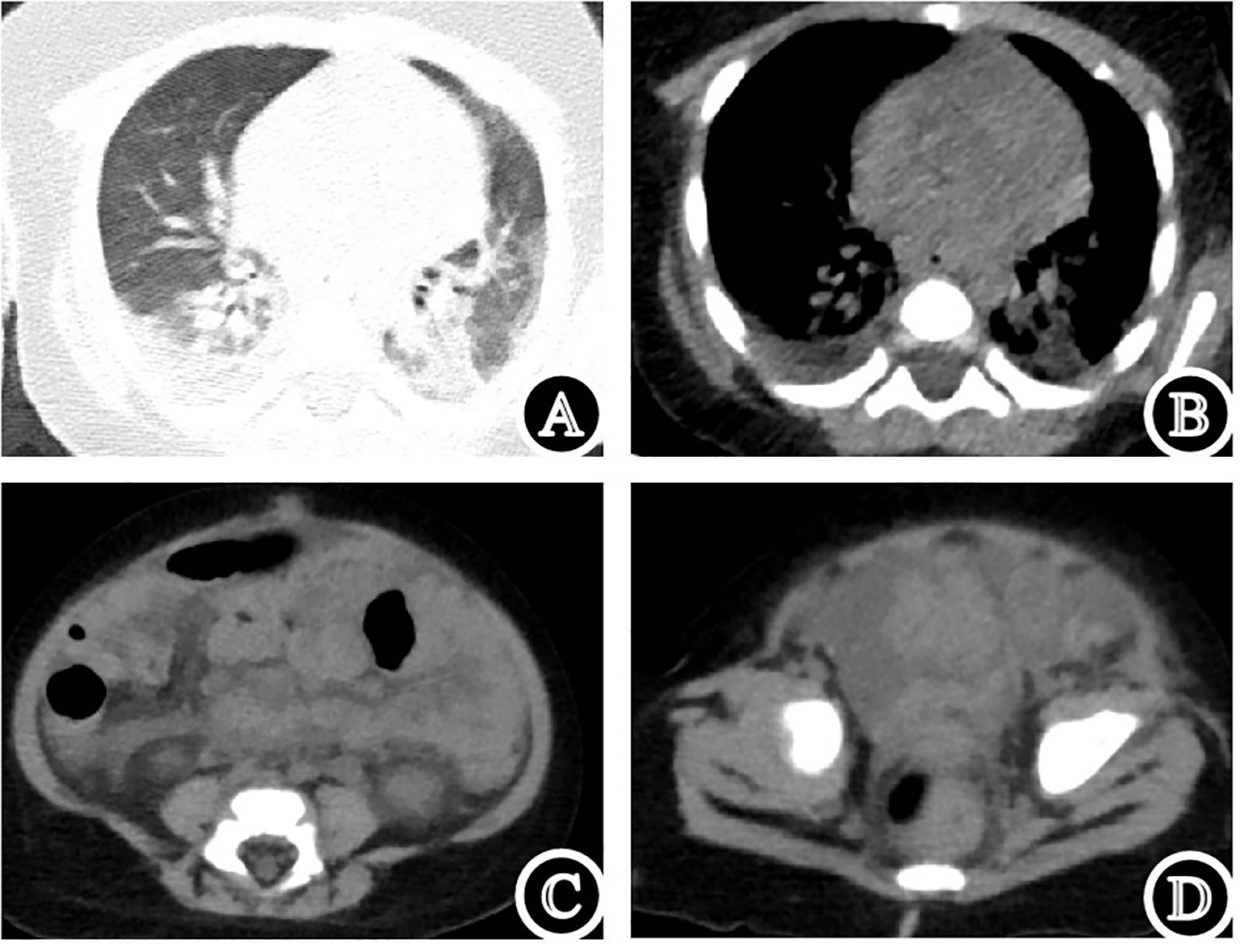
Clinical images of the patient. **(A, B)** Chest computed tomography (CT). **(C, D)** Abdominal and pelvic CT.

A 4-month-old male was admitted because of sepsis and severe pneumonia. Physical examination revealed a pustular rash over the body, and rupture with fluid exudation in the Bacillus Calmette-Guérin (BCG) vaccination site. Initial laboratory tests showed elevated inflammatory markers, with hypogammaglobulinemia (IgG 2.66 g/L, IgA <0.0667 g/L, IgM 0.0727 g/L) and lymphopenia (0.9 × 10^9^/L) (**Figure 1B**). Lymphocyte subset analysis showed absent T lymphocytes (including CD4^+^ and CD8^+^ subsets), with NK and B lymphocyte counts of 3/μL and 753/μL, respectively (**Figure 1A**). Chest computed tomography (CT) showed bilateral consolidations with pleural effusions (**Figures 2A, B**). Abdominal and pelvic CT revealed peritoneal and pelvic effusions (**Figures 2C, D**). Bone marrow examination showed active hyperplasia with granulocytic hyperplasia, erythroid hypoplasia, megakaryocytic dysplasia, and absent T lymphocytes (**Figure 1C**). Mycobacterium tuberculosis complex was detected in sputum by Xpert MTB/RIF^®^ assay, with rifampicin resistance detected in both sputum and ascitic fluid samples. Additionally, the Mycobacterium bovis complex was detected in the blood using metagenomic next-generation sequencing.

**Figure 3:**
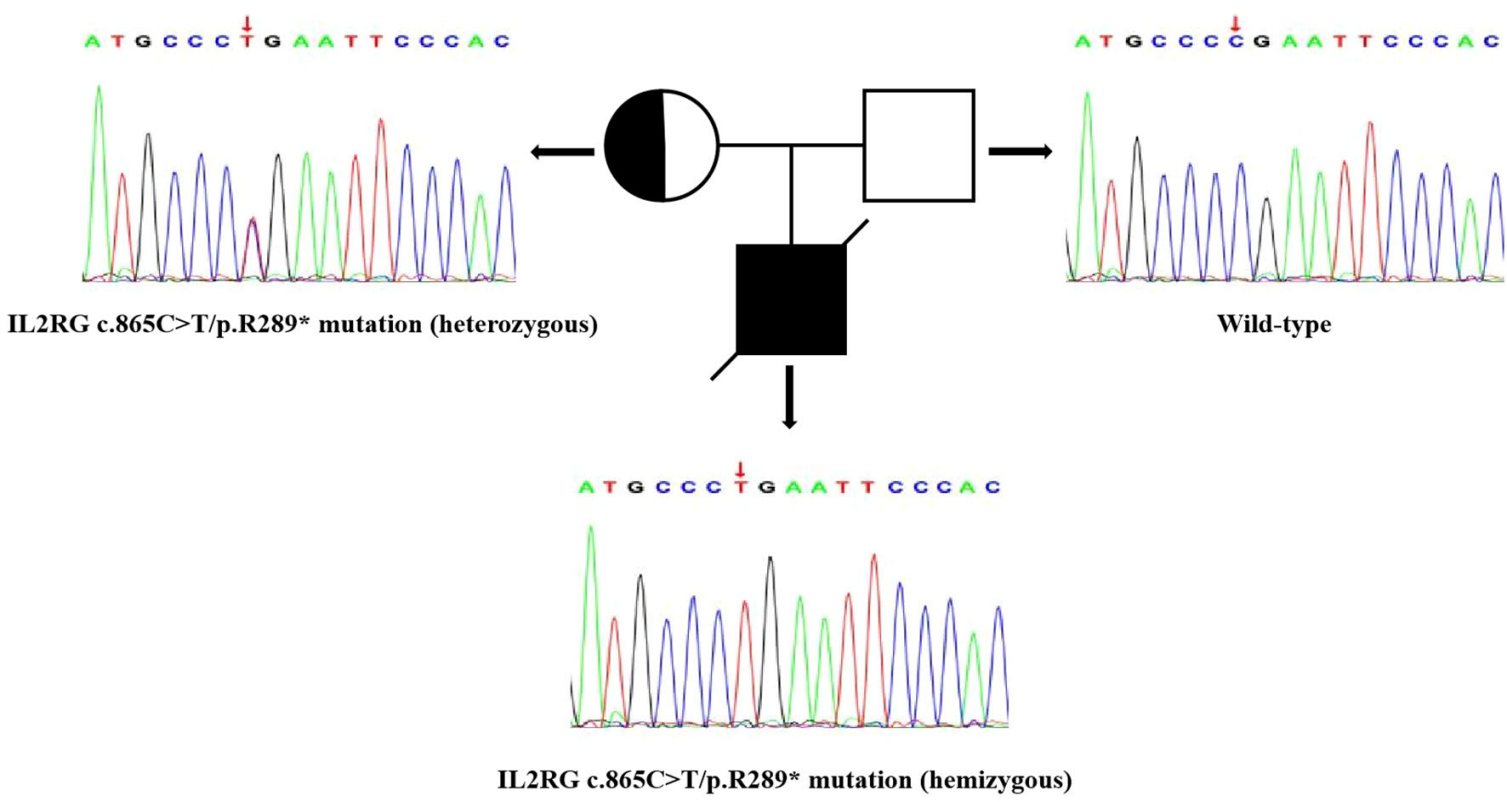
Sanger chromatogram of IL2RG gene mutation detected in the present case and his family. Squares = males; circles = females; solid squares with line across = deceased patient; bicolor circle = carrier.

Cellular and humoral immunity indices indicated combined immunodeficiency, and therefore genetic testing was performed on the patient and his parents. Whole-exome and Sanger sequencing identified a point mutation (c.865C>T) in exon 7 of the IL2RG gene (chrX:g.70328186), resulting in a nonsense mutation at the protein level (p.Arg289Ter) in the IL2RG protein (**Figure 3**). Currently, there are no reports in the literature on this rare nonsense mutation, either domestically or internationally. Experimental data suggested that this mutation was inherited from the patient’s mother (heterozygous state), while the father did not carry this mutation.

The patient was treated with broad-spectrum antibiotics, anti-tuberculosis therapy, and respiratory support. Detailed laboratory indicators and their dynamic changes are shown in **Figure 1B**.

On the 33rd day of admission, the patient’s hemoglobin level dropped to 54 g/L and he accidentally received non-irradiated leucoreduced erythrocyte suspension. One day later, lymphocyte subset analysis showed a mild increase in T/NK lymphocytes and a decrease in B lymphocytes (**Figure 1A**). Three days later, the patient became febrile (39.5°C), and the liver was palpable 6 cm below the right costal margin. Seven days later, the absolute blood eosinophil counts began to increase, reaching a peak on day 29 after infusion (**Figure 1B**). Sixteen days later, disseminated erythematous squamous skin rash appeared all over the body, and acute diarrhea developed concurrently. Twenty-four days later, the T lymphocyte counts increased to 1631 cells/μL. Twenty-eight days later, bone marrow re-examination showed T lymphocytes reappearance, B/NK lymphocytes reduction, eosinophilic hyperplasia, and severe erythroid hypoplasia (**Figure 1D**). Thirty days after infusion, a downward trend developed in all peripheral blood cell counts (**Figure 1B**).

The increased number of T lymphocytes in both peripheral blood and bone marrow were not consistent with the characteristics of SCID. Fever, skin rash, severe diarrhea, hepatomegaly, and the history of blood transfusion suggested TA-GVHD. Therefore, high-resolution Human Leukocyte Antigen (HLA) typing was performed on peripheral blood samples, and 10-20% interference from third-party HLA gene was detected. A skin biopsy was planned for confirmation, but the parents chose to terminate treatment and the child was discharged on the 69th day of admission. Unfortunately, the patient died after discharge.

## 3 Systematic review

We searched the database PubMed through July 2025 for studies involving SCID and TA-GVHD, and identified a total of 16 patients, including 1 from our case report (**Table 1**). The median age at diagnosis was 4.6 months, and there was a predominance of male patients (75%). All patients received non-irradiated blood components, and all developed TA-GVHD with a median onset time of 7 days, manifesting as rash (n=14), diarrhea (n=8), hepatomegaly (n=8), fever (n=8), pancytopenia (n=7), and increased peripheral blood lymphocyte percentage (n=5). The mortality rate was 93.75% (n=15), with a median survival time of 18 days. The only survivor was a 12-year-old boy who received an HLA-compatible bone marrow graft.

**Table 1:** TA-GVHD in patient with SCID.

No.	Sex	Age	Manifestations before transfusion	Laboratory indicators before transfusion	Blood transfused	Manifestations after transfusion	Laboratory indicators after transfusion (Units)	Onset time	Outcome	Ref.
1	F	4m	Soft tissue infection, fever, pneumonia, sepsis, rash.	• Blood cells: WBC: 11.3×10^9^/L; Lymphocytes: 0.9×10^9^/L; Eosinophils: 0.05×10^9^/L; • Immunoglobulins: IgG: 3.04 g/L; IgA: <0.0667 g/L; IgM: 0.265 g/L; • Lymphocyte subsets: CD3^+^: 0%; NK cells: 0.34%; B cells: 97.59%; • Bone marrow: active hyperplasia, granulocytic hyperplasia, erythroid hypoplasia, megakaryocytic dysplasia, absent T lymphocytes; • Genetic testing: Novel IL2RG c.865C>T (p.Arg289Ter) mutation;	Non-irradiated leucoreduced red blood cells	Fever, disseminated erythematous squamous skin rash, hepatomegaly, diarrhea, sepsis.	–	16 days	Death (35 days)	Our case
2	F	8m	Fever, vomiting, diarrhea, rash, Unhealed BCG vaccination site, hepatomegaly.	• Blood cells: WBC:8.4×10^9^/L; Lymphocytes: 2%; • Serum protein electrophoresis: Alpha_1_: 3.3 g/L; Alpha_2_: 11.0 g/L; Beta: 8.2 g/L; Gamma: 15.3 g/L;	Fresh, leukocyte-rich plasma	Diffuse erythematous macular rash, hepatomegaly, convulsions, respiratory distress, congestive heart failure.	• Blood cells (11 days after transfusion): Pancytopenia; Lymphocytes: 100%; • Bone marrow examination (12 days after transfusion): moderate number of histiocytic-appearing cells and no normal marrow elements	4 days	Death (14 days)	(9)
3	M	3/2m	Rash, Unhealed vaccination site, fever,	• Blood cells: WBC: 3.5×10^9^/L; Lymphocytes: 20%; • Serum protein electrophoresis: Alpha_1_: 5 g/L; Alpha_2_: 14.0 g/L; Beta: 9g/L; Gamma: 3 g/L; • Immunoglobulins: IgA: 0.05 g/L; IgM: < 0.05 g/L; IgG: 4.1 g/L;	Whole Blood	Fever, hepatosplenomegaly, diffuse erythematous macular rash, hypertonia, opisthotonos, respiratory distress, apnea.	• Blood cells (13 days after transfusion): Pancytopenia; • Bone marrow examination (15 days after transfusion): only a few large histiocytes	10 days	Death (17 days)	(9)
4	–	4m	Respiratory distress, anemia, hypoxia, Pneumocystis carinii pneumonia.	–	Non-irradiated Red Blood Cells	Rash, disseminated intravascular coagulation hypotension, arrhythmias, cardiac arrest.	• Blood cells: thrombocytopenia	–	Death	(14)
5	M	3m	Diarrhea, pneumonia.	• Blood cells: WBC: 4.075×10^9^/L; Lymphocytes: 33%; • Serum protein electrophoresis: Gamma: absent; • Immunoglobulins: IgA: 0 g/L; IgM: 0 g/L; IgG: <2.75 g/L;	Whole Blood	Diffuse morbilliform rash; Fever, pneumonia, diarrhea, edema, jaundice, sepsis.	• Blood cells: Lymphocytes:78% (14 days after transfusion)-90% (15 days after transfusion)-94% (16 days after transfusion)	10 days	Death (19 days)	(15)
6	M	11m	Fever, rash, hepatomegaly.	–	Whole Blood	Diffuse erythematous morbilliform rash, hepatosplenomegaly, diarrhea, edema, progression and necrosis of vaccinia lesions, deepening lethargy, abdominal distension, melena.	• Blood cells: 6 days after transfusion: WBC: 15×10^9^/L; Lymphocytes: 6%; 28 days after transfusion: Pancytopenia; • Serum protein electrophoresis (6 days after transfusion): Alpha_1_: 5 g/L; Alpha_2_: 12.0 g/L; Beta: 6 g/L; Gamma: 2 g/L; • Immunoglobulins (6 days after transfusion): IgA: <0.06 g/L; IgM: 0.14 g/L; IgG: 2.16 g/L; • Bone marrow examination: 6 days after transfusion: slight increase in myeloid elements, normal erythrocytic and megakaryocytic series, no plasma cells. 11 days after transfusion: myeloid hyperplasia, increased numbers of monocytoid and histiocytic cells.	10 days	Death (28 days)	(16)
7	M	8m	Oral moniliasis, weight gain cessation, persistent cough.	• Blood cells: WBC: 12.5×10^9^/L; lymphocytes: 19%; eosinophils: 11%; • Immunoglobulins: IgG: 20 g/L; IgA: <4.7 g/L; IgM: 4 g/L; • Bone marrow: Normal cellularity, normoblastic hyperplasia, eosinophilia, lymphopenia, no plasma cells;	Mother’s Blood	Fever, reticular erythematous macular eruption, generalized edema, anemia, congestive heart-failure, pulmonary distress, coma.	• Blood cells: 7 days after transfusion: WBC: 18×10^9^/L; later: anemia, neutropenia, normal lymphocyte-count; • Bone marrow examination: plasma cells can be found;	3 days	Death (15 days)	(17)
8	M	5m	Fever, dyspnea, cyanosis, weight loss, hepatomegaly, congestive heart failure, bronchitis, pneumonia.	• Blood cells: lymphocytes: low; • Serum protein electrophoresis: Gamma: 2.3%; • Immunoglobulins: absent;	Whole blood	–	–	–	Death (12 days)	(18)
9	F	3m	Vomiting, fever, weakness, pallor, jaundice, hepatosplenomegaly, lymphadenopathy, anemia.	• Blood cells: WBC: 47.1×10^9^/L; lymphocytes: 23%; eosinophils: 1%;	Packed Red Cells	Vomiting, diarrhea, moist eczematous eruption, inflamed mouth and throat, dysphagia, low-grade fever, weight loss, rectal bleeding.	• Blood cells (4 days after transfusion): WBC: 4.1×10^9^/L; lymphocytes: 68%; eosinophils: 3%;	2 days	Death (35 days)	(19)
10	M	12y	Watery diarrhea, conjunctivitis, whooping cough, German measles, varicella, bacterial septicemia, Candida infection.	–	–	Weight loss, exfoliative enteropathy, scaly skin rash, hepatomegaly.	–	–	Alive (HLA compatible Bone marrow graft)	(20)
11	M	3m	Fever, diffuse morbilliform rash, cough, severe diarrhea, dehydration, generalized edema, sclerema.	• Blood cells: WBC: 1.4×10^9^/L; Lymphocytes: 99%;	Fresh Blood	Hepatomegaly, Severe diarrhea, electrolyte imbalance, hyperbilirubinemia, E. coli septicemia.	• Blood cells (6 days after transfusion): Pancytopenia; WBC: 0.05×10^9^/L; • Serum protein electrophoresis (6 days after transfusion): Alpha_1_: 13%; Alpha_2_: 9%; Beta: 8%; Gamma: 4%; • Immunoglobulins (6 days after transfusion): IgG/IgM: Trace; IgA: Diminished; • Bone marrow examination: few large histiocytes, occasional medium-sized lymphocyte, few red cells.	6 days	Death (18 days)	(21)
12	M	4m	Diarrhea, hepatosplenomegaly, cardiomegaly, neurological symptoms, EEG changes.	• Blood cells: WBC: 10.2×10^9^/L; Lymphocytes: 23%; • Immunoglobulins: IgG/IgA/IgM: normal range;	Whole Blood, Packed Red Cells	Maculopapular rash, pulmonary edema, left hemiparesis, convulsions, respiratory failure.	• Blood cells: Eosinophils: No increased;	10 days	Death (18 days)	(22)
13	M	7m	Unhealed vaccination site, oral moniliasis, papules, diarrhea, semi-consciousness, severe dehydration, ulcers.	• Blood cells: WBC: 10×10^9^/L; Lymphocytes: 23%;	Whole Blood	Diarrhea recurred, erythematous rash.	–	6 days	Death (9 days)	(23)
14	F	18m	Diarrhea, progressive anemia, recurrent infections.	• Immunoglobulins: IgG: initially low then increased; IgA: low;	Whole Blood	Fever, macular erythematous rash, generalized subcutaneous edema, lymphadenopathy.	• Blood cells: Pancytopenia; Lymphocytes: Increased; • Immunoglobulin: increased;	7 days	Death (15 days)	(24)
15	M	2m	Pneumonia.	–	Non-irradiated Erythrocytes	Fever, generalized maculopapular rash, hepatomegaly, diarrhea, bone marrow aplasia.	• Blood cells: 7 days after transfusion: WBC: 200×10^9^/L; 15 days after transfusion: pancytopenia; • Immunoglobulins (7 days after transfusion):: IgG: 1.73 g/L; IgM: <0.168 g/L; IgA: <0.254 g/L; • Lymphocyte subsets (7 days after transfusion):: CD3^+^: 0.53%; CD4^+^: 2.3%; CD8^+^: 21.4%;	7 days	Death (47 days)	(8)
16	M	13m	Recurrent otitis media and respiratory infections.	–	Non-irradiated Erythrocytes	Fever, disseminated erythematous squamous skin rash, hepatomegaly, oral mucositis, diarrhea, multiorgan failure.	• Blood cells: 9 days after transfusion: WBC: 1200×10^9^/L; Later: pancytopenia; • Immunoglobulins (9 days after transfusion):: IgG: <1.46 g/L; IgM: 0.58 g/L; IgA: <0.218 g/L; • Lymphocyte subsets (9 days after transfusion):: CD3^+^: 0.9%; CD4^+^: 0.5%; CD8^+^: 0.3%; Hb: 70 g/L;	9 days	Death (99 days)	(8)

TA-GVHD, Transfusion-associated graft-versus-host disease; SCID, severe combined immunodeficiency; m, months; y, years; WBC, white blood cells; IgG, Immunoglobulin G; IgA, Immunoglobulin A; IgM, Immunoglobulin M; M, male; F, female; IL2RG, Arg289Ter; Arg, Arginine; Ter, Termination.

## 4 Discussion

In this report, we present a 4-month-old male infant with SCID caused by a novel and rare nonsense mutation in IL2RG who developed TA-GVHD following transfusion of non-irradiated leucoreduced red blood cells. Moreover, we reviewed and summarized the clinical characteristics and laboratory findings from previously reported cases of children with SCID who developed TA-GVHD.

The IL2RG gene is situated on the human X chromosome at position q13.1. It consists of 8 exons and spans 4,145 base pairs, encoding the gamma chain of the interleukin-2 receptor (25). The protein product is crucial for several cytokine receptors, including IL-2, IL-4, IL-7, IL-15, and IL-21,which regulate the differentiation and development of T lymphocytes, NK lymphocytes, and other cells (25). Mutations in IL2RG impair cytokine signaling, which arrests T lymphocyte development and leads to B lymphocyte dysfunction (12). Consequently, this results in combined humoral and cellular immune deficiencies, known as X-SCID.

The ClinVar and dbSNP databases catalog over 200 pathogenic mutations in IL2RG, including missense, nonsense, frameshift, and splice site mutations. In our report, the novel nonsense mutation [NM_000206.3(IL2RG): c.865C>T, p.(Arg289Ter)] is located in exon 7 of IL2RG. This mutation is located within the extracellular ligand-binding domain of IL2RG and was predicted to be deleterious by both Sorting Intolerant From Tolerant and Polymorphism Phenotyping v2. It introduces a premature termination codon at position 289, resulting in a truncated protein lacking critical functional domains, thereby abrogating IL-2, IL-7, and other related cytokine signaling (25). The predicted deleterious effect and mechanistic consequence of this mutation are consistent with those of previously reported pathogenic IL2RG nonsense mutations, thereby supporting its role in disease pathogenesis (26–28). According to the guidelines of the American College of Medical Genetics and Genomics, combined with the clinical phenotype and family analysis of the patient, this mutation meets the criteria for PVS1+PM2+PS3-Supporting pathogenicity classification and is considered a potential pathogenic mutation (29, 30).

GVHD is the clinical manifestation of the graft-versus-host reaction in man, which occurs following the infusion of immunocompetent cells into a recipient who is incapable of rejecting these cells (5). As a specific subtype of GVHD, transfusion-associated graft-versus-host disease (TA-GVHD) is a rare but fatal complication linked to blood transfusion, arising from viable donor lymphocytes in transfused blood components (9). SCID is a risk factor for TA-GVHD due to impaired elimination of alloreactive lymphocytes (3, 8, 31). In these patients, transfused T cells engraft, proliferate, and destroy recipient tissues expressing HLA class II antigens, including skin, liver, gastrointestinal tract, and bone marrow (32). The disease is fulminant and rapidly fatal, with nearly all patients with TA-GVHD dying of sepsis and multiple organ failure (4).

Therapeutic use of immunosuppressive agents including corticosteroids, cyclosporine, and tacrolimus has been reported for TA-GVHD (9, 22, 24). Despite this, the overall survival of patients with TA-GVHD has not been significantly improved by these interventions. In our literature review, only one patient survived, who received an HLA-compatible bone marrow graft. Therefore, early identification of TA-GVHD may provide a critical window to strategize patient management and potentially improve clinical outcomes. However, the above-mentioned symptoms of TA-GVHD are nonspecific and occur one to two weeks after a blood transfusion (8). In our case, we found that the engraftment of donor T lymphocytes in the recipient could be demonstrated by peripheral blood lymphocyte count as early as 1 day after blood transfusion, which may potentially offer a reference for early identification.

In our case, the changes in eosinophils in both peripheral blood and bone marrow, unreported previously in patients with SCID who develop TA-GVHD, deserve further investigation. Previous studies confirmed the inflammation-mediating role of eosinophils in the pathogenesis of GVHD, and eosinophils has been proposed as a biomarker for chronic GVHD (33–35). However, recent studies revealed an association between eosinophils and acute GVHD (aGVHD). and eosinophilia often precedes the development of aGVHD (33). Elevated absolute and relative eosinophil counts are demonstrated in patients with aGVHD, and eosinophilia often precedes the development of aGVHD in this patients (33, 35). Furthermore, in patients with aGVHD, blood eosinophils exhibit distinct activated phenotypes, characterized by increased expression of CD69, CD23, CD49d, and CD54 compared to patients without GVHD (33). Specifically, these studies link eosinophils to acute GVHD via Th2 activation and IL-5-mediated eosinophil production, activation, and survival (32). In our case, the increased blood eosinophil counts occurring after T lymphocyte proliferation may be associated with T cell-mediated inflammation, though this requires further investigation.

Since there is no satisfactory therapy for TA-GVHD, emphasis should be prevention (5). Irradiation of cellular blood components at doses of 2500–5000 rad represents the cornerstone strategy for preventing TA-GVHD by inhibiting the proliferation of donor T lymphocytes (5). Furthermore, leukocyte-depleted red blood cells and platelets should be employed to reduce the load of immunocompetent donor lymphocytes (22). Irradiation is required even for HLA-matched blood products from related donors, as shared haplotypes between the donor and recipient increase the risk of donor lymphocyte engraftment (9). Fresh whole blood and plasma should be avoided wherever possible, as these products contain viable lymphocytes capable of inducing GVHD (24). Therefore, irradiated, leukocyte-depleted cellular components should be employed for immunodeficient patients.

In conclusion, we report a case of SCID carrying a novel IL2RG mutation that developed TA-GVHD and review the relevant literature, revealing the reference value of blood absolute lymphocyte and eosinophil counts for early identification of TA-GVHD. However, future studies are needed to confirm these findings.

## Data Availability

The original contributions presented in the study are included in the article/Supplementary Material. Further inquiries can be directed to the corresponding authors.
